# Emergence of highly resistant *Candida auris* in the United Arab Emirates: a retrospective analysis of evolving national trends

**DOI:** 10.3389/fpubh.2023.1244358

**Published:** 2024-01-12

**Authors:** Jens Thomsen, Najiba M. Abdulrazzaq, Abderrahim Oulhaj, Peter S. Nyasulu, Adnan Alatoom, David W. Denning, Fatima Al Dhaheri, the UAE AMR Surveillance Consortium, Godfred Antony Menezes, Carole Ayoub Moubareck, Abiola Senok, Dean B. Everett

**Affiliations:** ^1^Department of Environmental and Occupational Health and Safey, Abu Dhabi Publich Health Center, Abu Dhabi, United Arab Emirates; ^2^Department of Pathology and Infectious Diseases, Khalifa University, Abu Dhabi, United Arab Emirates; ^3^Al Kuwait Hospital Dubai, Emirates Health Services Establishment (EHS), Dubai, United Arab Emirates; ^4^Department of Epidemiology and Public Health, Khalifa University, Abu Dhabi, United Arab Emirates; ^5^Department of Global Health, Faculty of Medicine and Health Sciences, Stellenbosch University, Cape Town, South Africa; ^6^Department of Pathology, Sheikh Shakhbout Medical City, Abu Dhabi, United Arab Emirates; ^7^Manchester Fungal Infection Group, The University of Manchester, Manchester, United Kingdom; ^8^Department of Pediatrics, College of Medicine and Health Sciences, United Arab Emirates University, Al Ain, United Arab Emirates; ^9^Department of Medical Microbiology and Immunology, Ras Al Khaimah Medical and Health Sciences University, Ras Al Khaimah, United Arab Emirates; ^10^College of Natural and Health Sciences, Zayed University, Dubai, United Arab Emirates; ^11^College of Medicine, Mohammed Bin Rashid University of Medicine and Health Sciences, Dubai, United Arab Emirates; ^12^School of Dentistry, Cardiff University, Cardiff, United Kingdom; ^13^Biotechnology Research Center, Khalifa University, Abu Dhabi, United Arab Emirates; ^14^Infection Research Unit, Khalifa University, Abu Dhabi, United Arab Emirates

**Keywords:** *Candida auris*, surveillance, healthcare-associated infections, antifungals, antimicrobial-resistance, UAE, MENA

## Abstract

**Introduction:**

The Centers for Disease Prevention and Control lists *Candida auris*, given its global emergence, multidrug resistance, high mortality, and persistent transmissions in health care settings as one of five urgent threats. As a new threat, the need for surveillance of *C. auris* is critical. This is particularly important for a cosmopolitan setting and global hub such as the United Arab Emirates (UAE) where continued introduction and emergence of resistant variant strains is a major concern.

**Methods:**

The United Arab Emirates has carried out a 12 years of antimicrobial resistance surveillance (2010–2021) across the country, spanning all seven Emirates. A retrospective analysis of *C. auris* emergence from 2018–2021 was undertaken, utilising the demographic and microbiological data collected via a unified WHONET platform for AMR surveillance.

**Results:**

Nine hundred eight non-duplicate *C. auris* isolates were reported from 2018–2021. An exponential upward trend of cases was found. Most isolates were isolated from urine, blood, skin and soft tissue, and the respiratory tract. UAE nationals nationals comprised 29% (*n* = 186 of 632) of all patients; the remainder were from 34 other nations. Almost all isolates were from inpatient settings (89.0%, *n* = 809). The cases show widespread distribution across all reporting sites in the country. *C. auris* resistance levels remained consistently high across all classes of antifungals used. *C. auris* in this population remains highly resistant to azoles (fluconazole, 72.6% in 2021) and amphotericin. Echinocandin resistance has now emerged and is increasing annually. There was no statistically significant difference in mortality between *Candida auris* and *Candida* spp. (non-auris) patients (*p*-value: 0.8179), however *Candida auris* patients had a higher intensive care unit (ICU) admission rate (*p*-value <0.0001) and longer hospital stay (*p* < 0.0001) compared to *Candida* spp. (non-auris) patients.

**Conclusion:**

The increasing trend of *C. auris* detection and associated multidrug resistant phenotypes in the UAE is alarming. Continued *C. auris* circulation in hospitals requires enhanced infection control measures to prevent continued dissemination.

## Introduction

Invasive candidiasis which encompasses *Candida* bloodstream infections and deep-seated candidiasis is a significant cause of morbidity and mortality (1–6), and remains a significant healthcare-associated problem in several countries (7, 8). Within the candidemia grouping, the first known case of *Candida auris* was in an ear infection in Japan in 2009 (9). *C. auris* has now become a major public health threat, due to its propensity for horizontal transmission (10–13) and its continued nosocomial spread in long-term and acute care healthcare facilities (6, 11, 14).

*C. auris* has quickly developed into a global concern and cemented its place as a superbug within just a decade after its first isolation in 2009 (9). Since its emergence, it has been identified in hospitals across five continents, particularly increasing in incidence during the COVID-19 pandemic (4, 15, 16). The role played by the coronavirus disease (COVID-19) pandemic in this increase is difficult to ascertain, while restricted travel may have decreased the risk of importation of *C. auris*, difficult-to-control outbreaks of *C. auris* have continued to be reported in units caring for COVID-19 patients worldwide (17–20). *C. auris* presents diagnostic challenges because of difficulty in identifying strains using common microbiological procedures and challenges in treatment given its resistance to multiple anti-fungal agents, including azoles, echinocandins, and polyenes, making it a critical antibiotic resistance threat (21, 22).

*C. auris* is now listed among five urgent threats defined in the U.S. Centers for Disease Prevention and Control’s (CDC) 2019 Antibiotic Resistance Threats Report due to its global emergence, multidrug resistance, high mortality, and persistent transmissions in health care settings (9, 10, 23–26). A systematic review and meta-analysis that included cases between 2009 and 2019 from different countries reported an average crude mortality of 45% (95% CI: 39–51%) for *C. auris* bloodstream infections (21). However, mortality attributable to *C. auris* remains unclear. The vast majority of strains are fluconazole resistant, with variable proportions resistant to amphotericin B, echinocandins and flucytosine. Reports of antifungal susceptibility data from different geographic locations are varied and some *C. auris* strains exhibit elevated MICs for three major classes of antifungal drugs. The CDC has suggested tentative breakpoints, and these have been used in most studies, EUCAST and CLSI have yet to recommend clinical breakpoints or epidemiological cut-offs (27–29).

An astonishing aspect in relation to the rapid emergence of *C. auris* is the simultaneous but independent appearance of genetically distinct clades on different continents (4, 15). The whole-genome sequence (WGS) analysis of clinical isolates of *C. auris* collected from South Asia (India/Pakistan), South Africa and East Asia (Korea/Japan) has shown four highly clonal phylogenetic and geographically distinct clades that have emerged seemingly independent of one another, specifically, the South Asian clade (clade I), the East Asian clade (clade II), the South African clade (clade III), and the South American clade (clade IV) (4, 15, 30). In 2018, a fifth clade, which is exclusively found in Iran (Iranian clade), was identified (10, 24, 31).

Antifungal resistance is widespread in *C. auris* in the South Asia clade I isolates. These isolates are resistant to fluconazole, variably resistant to amphotericin B, and also acquire resistance to echinocandins (32–35). *C. auris* South America clade IV includes isolates with variable resistance to amphotericin B (36, 37), while South Africa clade III isolates are frequently resistant to azoles antifungals (38). Multidrug resistant *C. auris* isolates to three major classes of antifungal agents have also emerged (10, 39, 40). This severely limits treatment options, making infection control and prevention in healthcare settings essential (5).

The global number of *C. auris* cases has been rapidly increasing in the past few years particularly in blood cultures from patients with serious underlying medical conditions and in hospitalized patients with invasive medical devices, such as urinary tract catheters and parenteral nutrition, who have also received broad-spectrum antibiotics (1, 3). Mortality in *C. auris*-associated infections has been reported from 33.3% to 100% worldwide (21), and more recent data has indicated a similar (high) mortality compared to other *Candida* bloodstream infections (41–43).

Since the time of its first isolation in Japan, *C. auris* infections have been reported from several countries including South Korea, Malaysia, Kenya, South Africa, India, Pakistan, Colombia, Venezuela, Panama, United States, Canada, China, Russia and Europe (21). Among 17 countries listed under the MENA region, invasive *C. auris* infections have only been reported from Kuwait in (44–46), Israel (3), Oman (47, 48), Saudi Arabia (49), United Arab Emirates (50), Iran (51) and Qatar (52, 53) to date. The real prevalence and epidemiology of *C. auris* remains unknown in this region.

### United Arab Emirates

Currently, the country hosts a population of nearly 10 million people of which 1 million are Emirati citizens, and the rest are mixed expatriates from various nationalities. The majority of this population resides in Abu Dhabi and Dubai, the two biggest Emirates of the seven that form the UAE (54). The first UAE report of *C. auris* was in a female patient with persistent candidemia who was admitted to Cleveland Clinic Abu Dhabi Hospital in 2018 (50). The patient had a protracted hospital stay over 1 year with several co-morbid conditions including chronic renal failure on hemodialysis, severe psoriasis, chronic atrial fibrillation and hypertension. During hospitalization the patient was admitted to intensive care unit (ICU) repeatedly and developed multiple infections (bloodstream infections, pneumonia, urinary tract infections) due to several bacterial and fungal pathogens. The patient deteriorated over the next month and died 3 months after the first isolation of *C. auris* from her blood (50). This has been the only reported case of *C. auris* in the UAE.

Here we present the first comprehensive UAE wide retrospective epidemiological analysis of all reported *C. auris* data to date. Thus this study aimed to investigate the trend in the incidence of *C. auris* over a 4 years period from 2018 to 2021.

## Methods

The UAE has been carrying out a national AMR surveillance program over the past 12 years (2010–2021). A retrospective study of emerging *C. auris* was conducted from 2018 to 2021, using data from the UAE national AMR surveillance program. This data is gathered through a unified WHONET platform (https://whonet.org/). Data collected included demographic and microbiological parameters from all participating centers across the country. The participating sites were managed by trained personnel who gathered AMR surveillance data from routine patient care and submitted it to the National AMR surveillance program. Data was generated, collected, cleaned and analyzed through the national AMR surveillance program as described by Thomsen et al. (55).

### Identification of *Candida auris*

*C. auris* identification was performed at the national AMR surveillance sites by medical professionals. *C. auris* isolates were identified and tested for antifungal susceptibility using mostly commercial, automated systems including VITEK^®^ (BioMérieux SA, Craponne, France), BD Phoenix^™^ (Becton Dickinson, New Jersey, United States), and MicroScan^™^ (Beckman Coulter, California, United States). A few laboratories used Sensititre YeastOne^™^ (Thermo Scientific, Massachusetts, United States) plates for susceptibility. Only one laboratory (out of 45 labs) relied on a manual API^®^ (Analytical Profile Index. BioMérieux SA, Craponne, France) system for identification, and only two labs conducted susceptibility testing by manual disc diffusion.

### Antimicrobial resistance trends in *Candida auris*

This was assessed by analysis of routine national level AMR surveillance data. This data, which covers a spectrum of AMR pathogens including *C. auris*, was obtained from across a network of 317 participating hospitals (*n* = 84), centers and clinics (*n* = 233), and 45 diagnostic laboratories in the country. These participating centers include primary, secondary and tertiary care facilities as well as public and private entities. All data are routinely collected and analysed using a unified platform (WHONET) and training on data collection is provided to ensure quality assurance, standardization and accuracy. The fully anonymized data includes demographic data (age, gender, nationality, hospital site/location etc.), clinical and microbiological data such as specimen source and antifungal susceptibility testing results. For the purpose of this analysis, we applied the CDC tentative breakpoints to determine susceptibility of our isolates (29). Resistance MIC breakpoints were as follows: fluconazole ≥ 32 µg/mL; amphotericin *B* ≥ 2; caspofungin ≥2; anidulafungin and micafungin ≥4.

### Data sources and statistical analysis

AMR data was extracted from the national AMR surveillance database. *p* < 0.05 were considered statistically significant. We performed three types of analyses. In the first analysis, binary logistic regression was used to model the proportion of positive *C. auris* among all reported infections. Estimates of this analysis provide evidence regarding the annual increase in the reported positive *C. auris* cases among all reported cases. In the second analysis, the binary logistic regression model was used to investigate the proportion of positive *C. auris* among reported *Candida* spp. cases only. Estimates of this model provide data regarding the annual increase in the reported positive *C. auris* cases among *Candida* spp. cases. One main limitation of the above two analyses is the possibility that the trend in positive *C. auris* cases over time could be due to a potential increase in the screening of *C. auris* over time. To adjust for this potential bias, the total number of tests performed to screen for *C. auris* should be used. Unfortunately, these metrics are not available in the database. To investigate this possibility, we conducted a large simulation study where different scenarios for the annual increase in the screening rate of *C. auris* are assumed (see Supplementary material for more details). For each hypothetical screening rate, a binary logistic regression model was fitted, and significance and direction of percentage change in *C. auris* reported. For all three analyses, odds ratio and corresponding 95% confidence intervals were derived, and provide indication of the change over time in the incidence of positive *C. auris* cases (increase, or decrease, or no change over time). A chi-square test was used to test the association between categorical variables including mortality and ICU admission. The weighted log rank test was used to assess differences in length of stay in hospital. Binary logistic regression analyses and chi-square test for data presented in tables was performed using the R software (R: The R Project for Statistical Computing, n.d.), chi-square test for mortality rate was performed using Epi Info^™^ for Windows v7.2.4.0.

### Overview of the UAE national AMR surveillance

**Figure 1:**
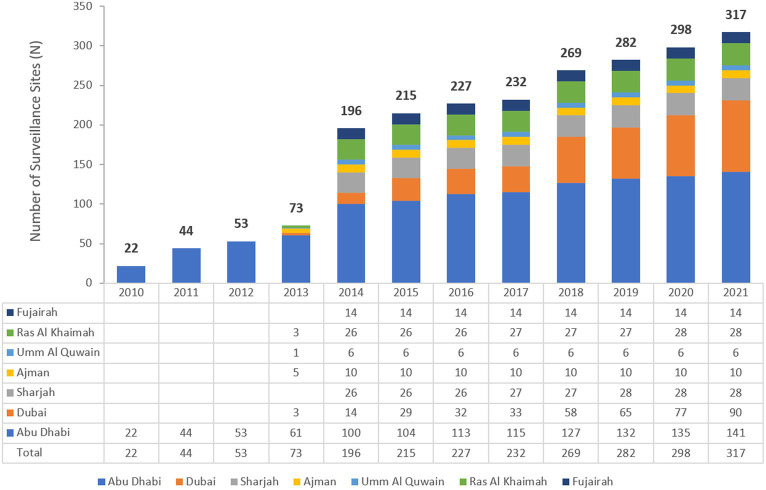
Number of reporting centers (2010–2021), by Year and Emirate.

The UAE national AMR surveillance was initiated in 2010 in the Abu Dhabi Emirate where 6 hospitals and 16 Centers/Clinics adopted the WHONET 2021 Software for AMR surveillance.^1^ Additional sites were recruited over the years, starting with only 22 participating sites in 2010, which is the first year during which the study started, and located only in the Emirate of Abu Dhabi to reach a total of 317 surveillance sites from the 7 Emirates, including 84 hospitals and 233 centres/clinics and representing all seven Emirates of the country in 2021. Figure 1 shows the distribution of surveillance sites for National AMR Surveillance program from 2010 to 2021.

## Results

### Demographic, clinical and health outcomes of *Candida auris*

**Figure 2:**
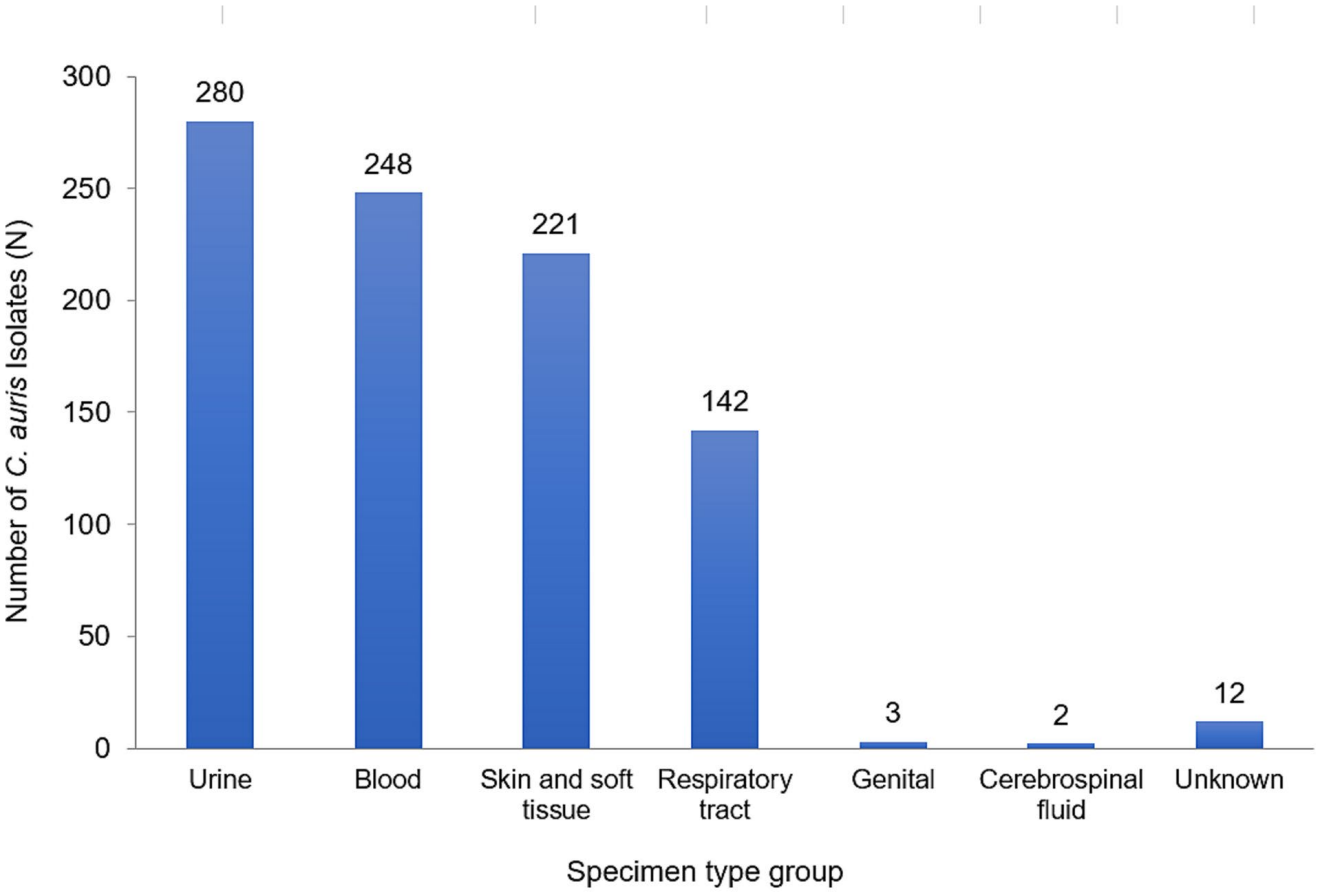
Distribution of *Candida auris* isolates/patients, by specimen type group, UAE, 2018–2021, *N* = 908.

A total of 908 non-duplicate *C. auris* isolates were reported from 2018–2021 (2018: *n* = 9; 2019: *n* = 93; 2020: *n* = 192; 2021: *n* = 614). Most of *C. auris* isolates were obtained from urine (280/908, 30.8%), blood (248/908, 27.3%) and skin and soft tissue (221/908, 24.3%). This was followed by respiratory tract (142/908, 15.6%), genital tract (3/908, 0.3%), and cerebrospinal fluid (CSF) specimens (2/908, 0.2%). *C. auris* was isolated across a broad range of sample types, showing widespread dissemination. Figure 2 shows the distribution of specimen types where *C. auris* was isolated from.

**Figure 3:**
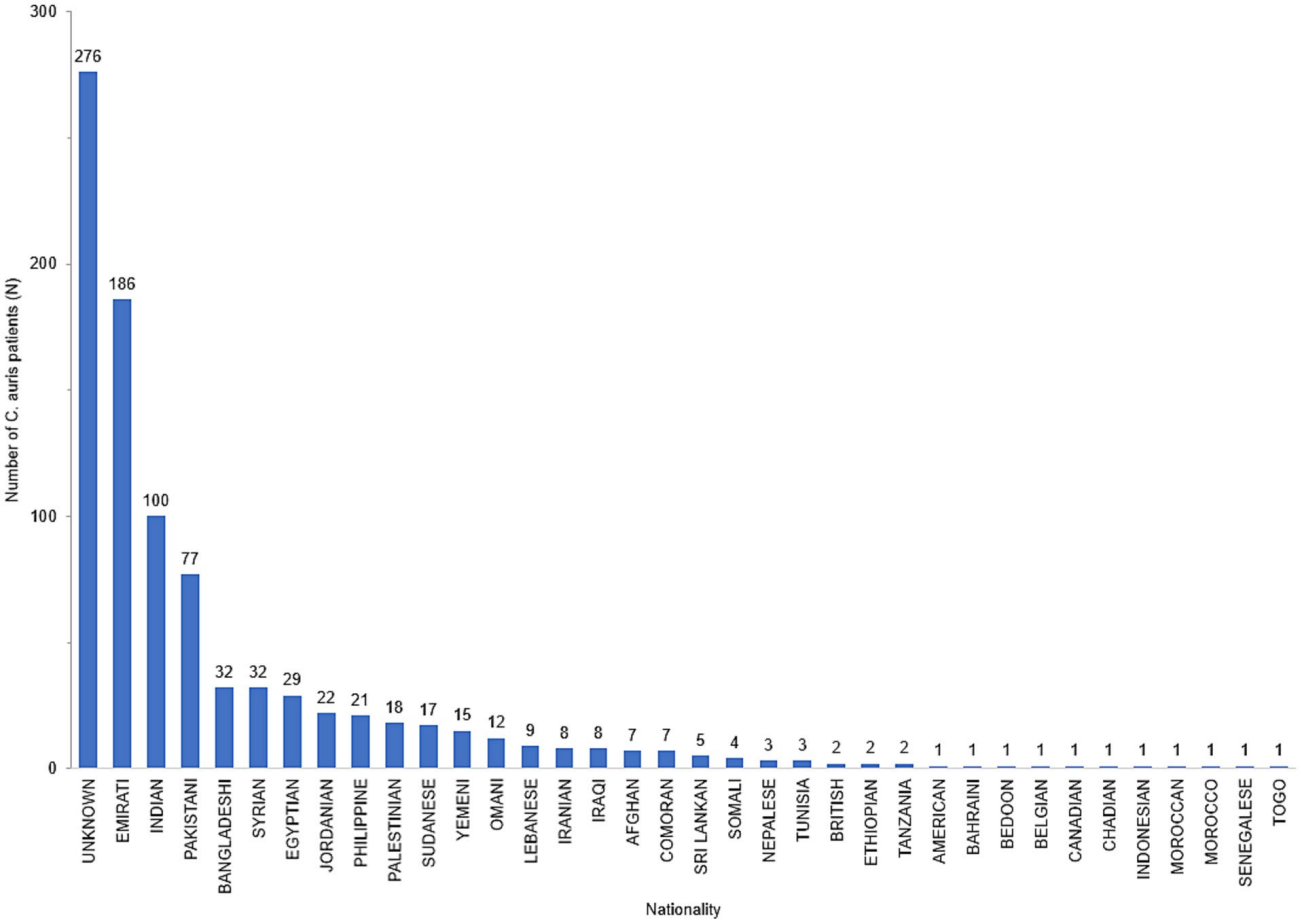
Distribution of *Candida auris* patients by Nationality, UAE, 2018–2021.

Data on nationality was available for 632 patients of whom 29.4% (*n* = 186) were UAE nationals and the remainder (70.6%) comprised of individuals from 34 other nationalities (Figure 3). The demographic distribution of the patients shows a heavily skewed distribution across inpatient settings (809/908, 89%) and predominantly ICU patients (414/908, 45.6%). It also revealed a male preponderance with majority of patients being in the adult age group (Table 1).

**Table 1:** Demographic distribution of *Candida auris* cases and *Candida* spp. (non-auris) patients.

Demographic	Category	*Candida auris* cases (*N* = 908)		*Candida* spp. (non-auris) cases (*N* = 21,818)		*p*-value
		*N*	%	*N*	%	
Gender	Male	474	52.2	5,539	25.4	<0.0001
	Female	224	24.7	13,439	61.6	
	Unknown	210	23.1	2,840	13.0	
Age group	Paediatric	7	0.8	689	3.2	0.0003
	Adult	666	73.4	17,500	80.2	
	Unknown	235	25.9	2,957	13.6	
Nationality	Emirati	186	20.5	5,669	26.0	<0.0001
	Non-Emirati	446	49.1	9,086	41.6	
	Unknown	276	30.4	7,064	32.4	
Patient location	ICU	414	45.6	3,905	17.6	
	Inpatient	395	43.5	5,763	26.0	<0.0001
	Outpatient	24	2.6	9,620	43.3	
	Unknown	75	8.3	2,911	13.1	
Emirate	Abu Dhabi	275	30.3	8,680	39.8	<0.0001
	Ajman	56	6.2	628	2.9	
	Dubai	214	23.6	7,610	34.9	
	Fujairah	6	0.7	245	1.1	
	Ras Al Khaimah	100	11.0	1,167	5.4	
	Sharjah	171	18.8	2,086	9.6	
	Umm Al Quwain	86	9.5	1,395	6.4	

#### Admission to intensive care unit

A total of 19,353 patients were associated with *Candida* spp. (non-auris) of whom 3,905 (20.2%) patients were admitted to ICU, while a total of 835 patients were associated with *Candida auris*, of whom 414 (49.6%) patients where admitted to ICU. The difference in ICU admission rate is statistically significant (*p* < 0.0001).

#### Length of stay

We performed a length of stay (LOS) analysis and assessed the differences in duration of hospitalization using a weighted log-rank test. We included data of patients for whom the date of admission and date of discharge was known. For those patients who were associated with *Candida* spp. (non-auris) (*n* = 4,912) the median length of stay was 14.0 days, while for those patients who were associated with *C. auris* (*n* = 140) the median length of stay was 33.5 days. The observed difference in length of hospitalization between patients associated with *C. auris* and non-*C. auris* spp. was statistically significant (chi square 64.1, *p* < 0.0001). Based on a total of *n* = 908 patients during the observation period (2018–2021), a total of 17,706 excess days of hospitalization were observed, attributable to *C. auris*. For the year 2021 only (*n* = 614 *C. auris* cases), a total of 11,973 excess hospitalization days were observed, attributable to *C. auris* (see Supplementary Figure S1). Kaplan-Meier curve: probability of longer hospitalization of *Candida auris* patients versus *Candida* spp. (non-auris) patients [UAE, 2010–2021].

#### Mortality rate

Analysis on a subset of patients for whom the health outcome was known was performed. A total of 5,694 patients were associated with *Candida* spp. (non-auris) of whom 1,503 patients died (mortality rate: 26.4%). A total of 171 patients were associated with *C. auris*, of whom 47 patients (mortality rate: 27.5%) died. The difference in proportion of those who died between *C. auris* patients and *Candida* spp. (non-auris) patients is not statistically significant (*p* = 0.818). Crude mortality rate for patients with *C. auris* isolates from blood cultures only was 22/61 (36.1%).

### Trend analysis of *Candida auris* among all reported infections: approach 1

**Figure 4:**
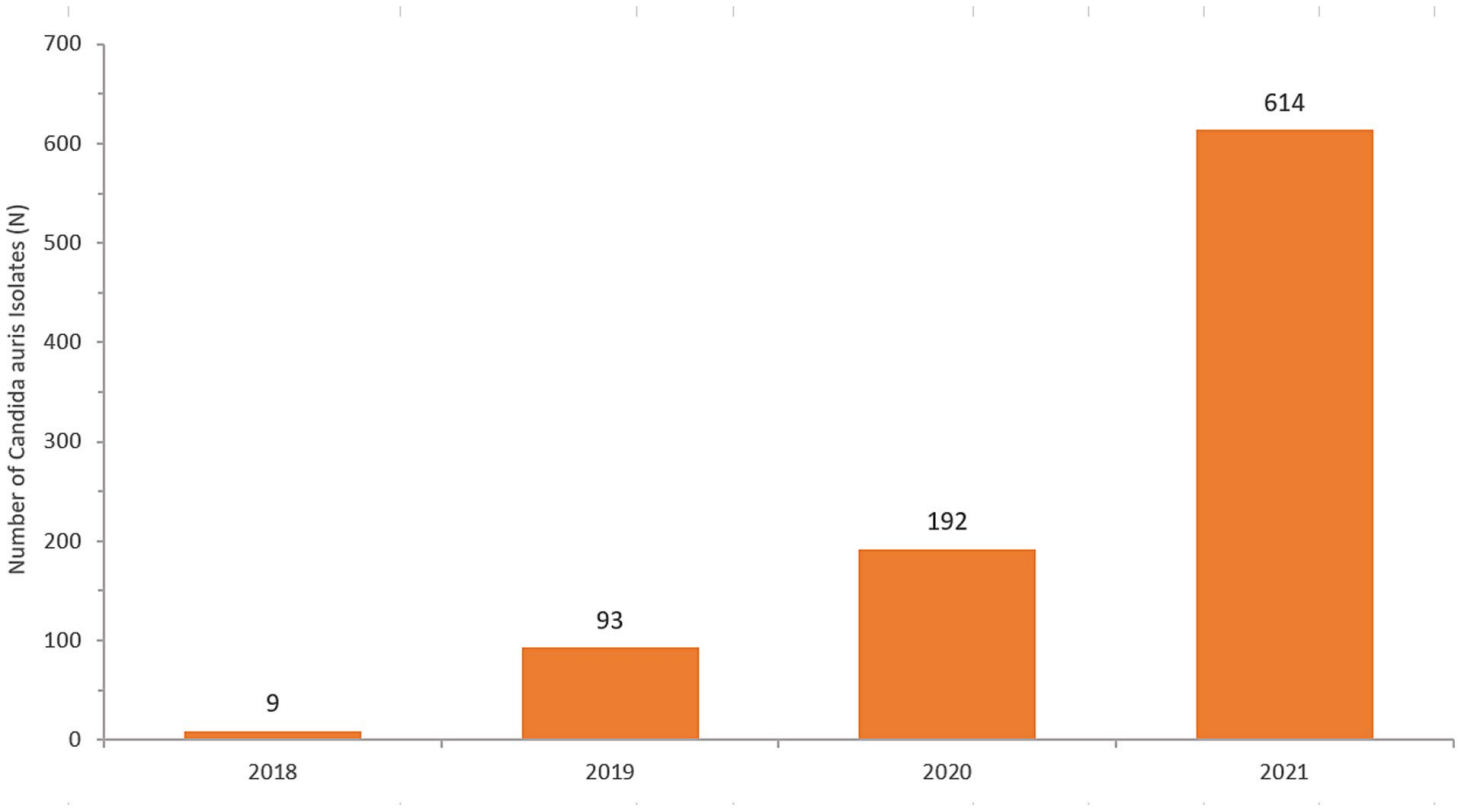
Number of reported *Candida auris* isolates (2018–2021).

Table 2 shows the number of cases of *C. auris* and the total number of national AMR surveillance cases reported from 2018 up to 2021, along with the proportion of positive *C. auris* cases for each year. Figure 4 shows the trend over time from 2018 to 2021.

**Table 2:** Number of cases of *C. auris* and the total number of cases reported from 2018 up to 2021.

Year	*C. auris* cases	Total UAE cases	Infection Rate
2018	9	95,315	0.0000944
2019	93	102,203	0.0009100
2020	192	91,097	0.0021076
2021	614	126,334	0.0048601

**Figure 5:**
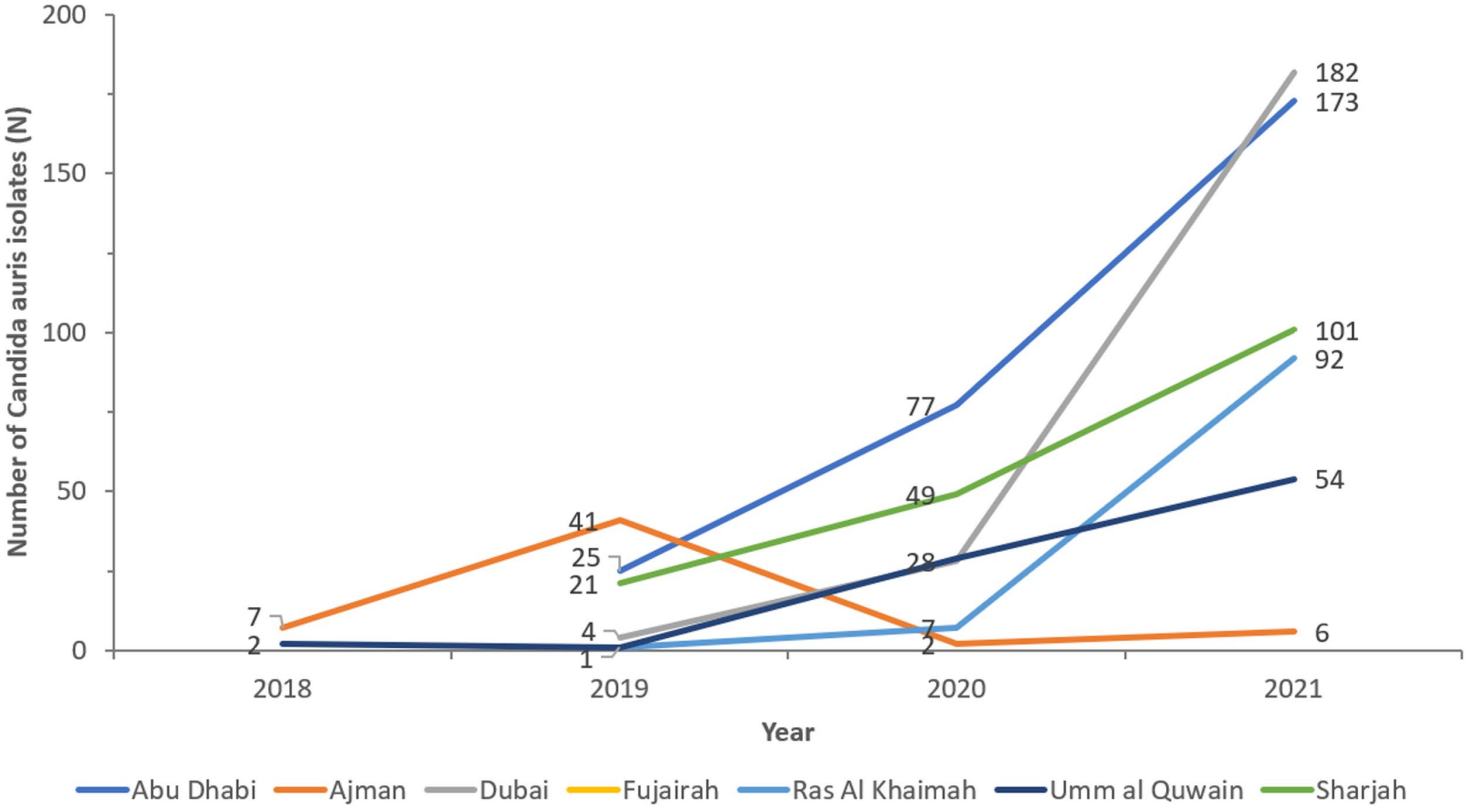
*Candida auris* isolate reporting trends over time by Emirate.

The cases show widespread distribution across all reporting sites and Emirates (Figure 5). Ajman and Umm Al Quwain first reported *C. auris* isolates in 2018. Emergence occurred in all other Emirates in 2019 and spread rapidly. Abu Dhabi and Sharjah have almost doubled cases annually. Dubai identified 4 cases in 2019 to 182 in 2021, representing a 4450% increase in cases in 2 years.

**Figure 6:**
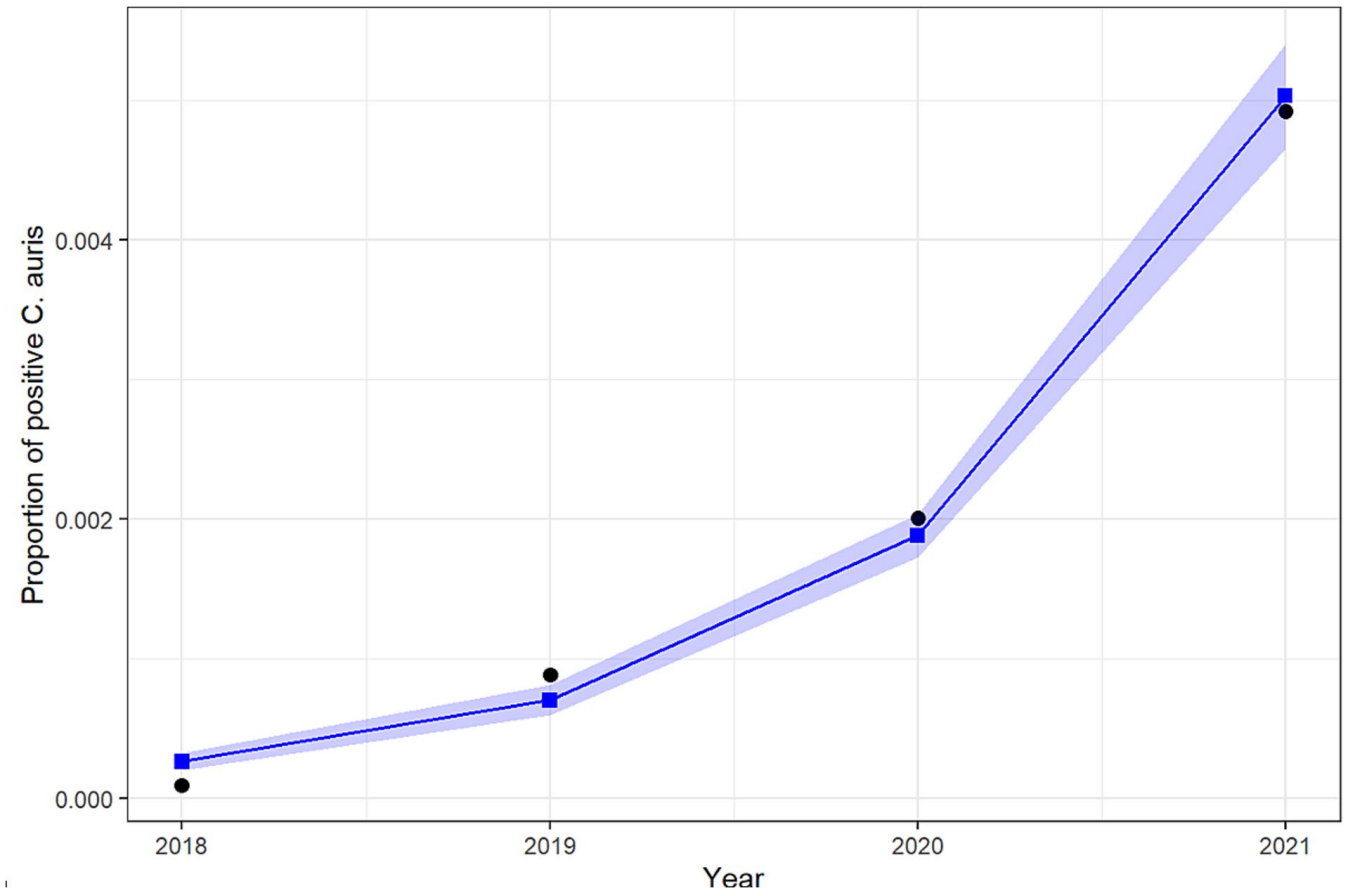
Predicted versus observed rate of positive *C. auris* among all infections.

The results of the logistic regression show a significant increase over the years in the odds of reporting positive *C. auris* cases among all reported cases. More specifically, the odds of reporting a positive *C. auris* cases increases by 161.5% (95% CI: 140.6–185.1%) each year from 2018 to 2021. Figure 6 shows the predicted versus the observed counts of positive *C. auris* cases derived from the fit of the binary logistic regression model.

### Trend analysis of *Candida auris* among all *Candida* spp. cases: approach 2

Table 3 shows the number of positive *C. auris* cases and the number of positive *Candida* spp. cases from 2018 up to 2021, along with the proportion of positive *C. auris* cases for each year.

**Table 3:** Cases of *C. auris* amongst all *Candida* spp. cases.

Year	*C. auris* cases	Total *Candida* cases	Infection rate
2018	9	2,278	0.0039508
2019	93	3,183	0.0292177
2020	192	3,829	0.0501436
2021	614	12,962	0.0473692

The results of the logistic regression show a significant increase over the years in the odds of reporting a positive *C. auris* case among *Candida* spp. cases. More specifically, the odds of reporting a positive *C. auris* case increases by 46.2% (95% CI: 35.1%–58.7.6%) each year from 2018 to 2021.

### Trend analysis of *Candida auris*: the simulation study: approach 3

**Figure 7:**
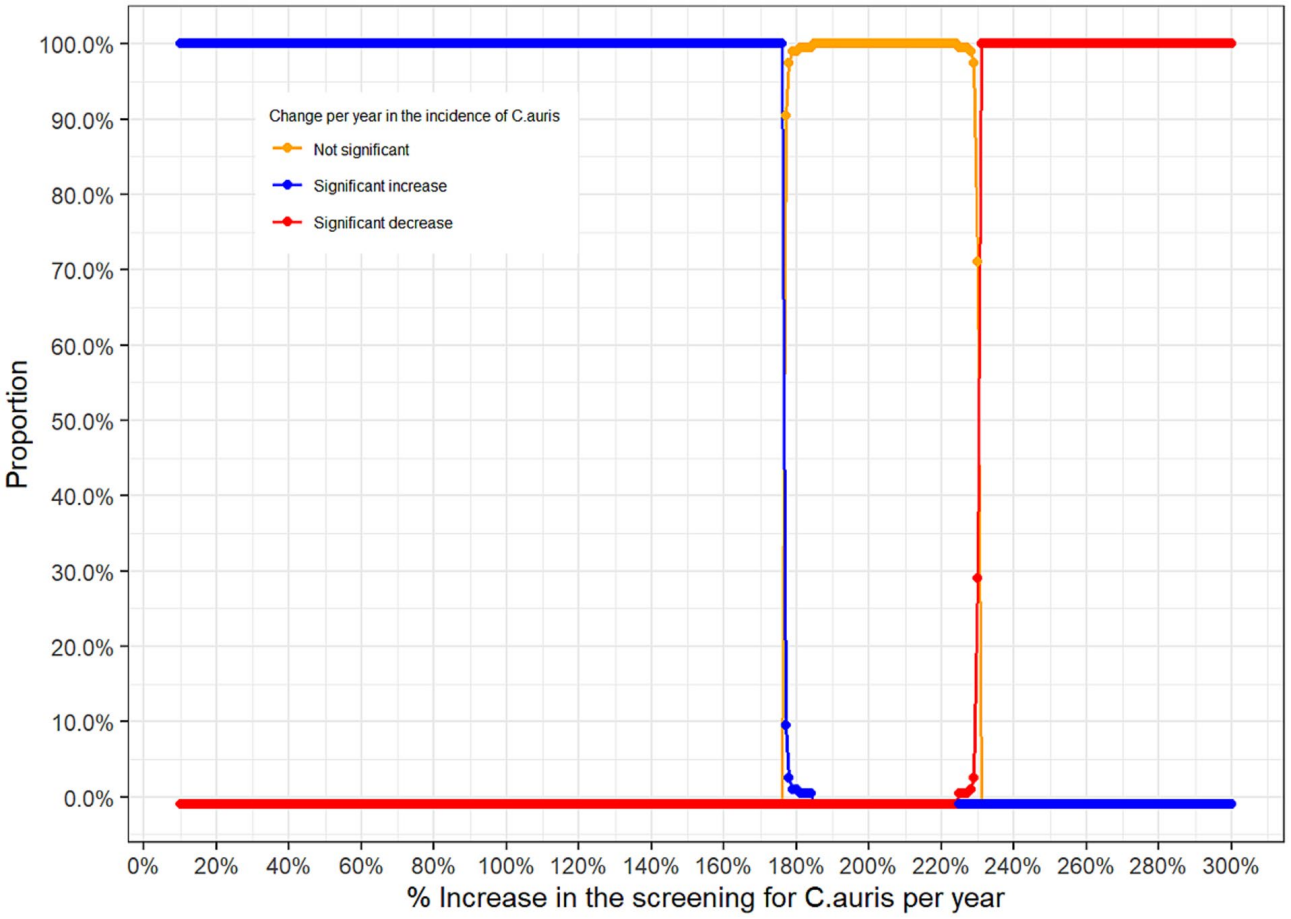
Proportion of significant results according to the hypothetical annual increase rate in the screening of *C. auris.*

One main limitation of the above two approaches to analyse the trend is the possibility that the trend in positive *C. auris* cases over time could be due to a potential increase in the screening of *C. auris* over time. To adjust for this potential bias, and due to the non-availability of the total number of tests performed to screen for *C. auris*, we conducted a large simulation study where different scenarios for the yearly increase in the screening rate of *C. auris* were assumed. Figure 7 provides, for each hypothetical annual increase in the screening rate of *C. auris*, the proportion of results with non-significant change, significant increase and significant decrease in the incidence of *C. auris* over time.

From the simulation study above, one can see that positive *C. auris* cases observed over the 4 years reflect a statistically significant increase in the incidence of *C. auris* over time if the annual increase in the screening for *C. auris* does not exceed 176% (blue curve). If the annual increase in the screening for *C. auris* lies between 177% and 225% then the trend observed is not statistically significant (orange curve), however, if the annual screening rate was above 225% then the positive *C. auris* cases observed over the 4 years reflect a statistically significant decrease in the incidence of *C. auris* over time (red curve).

### Antifungal resistance

**Figure 8:**
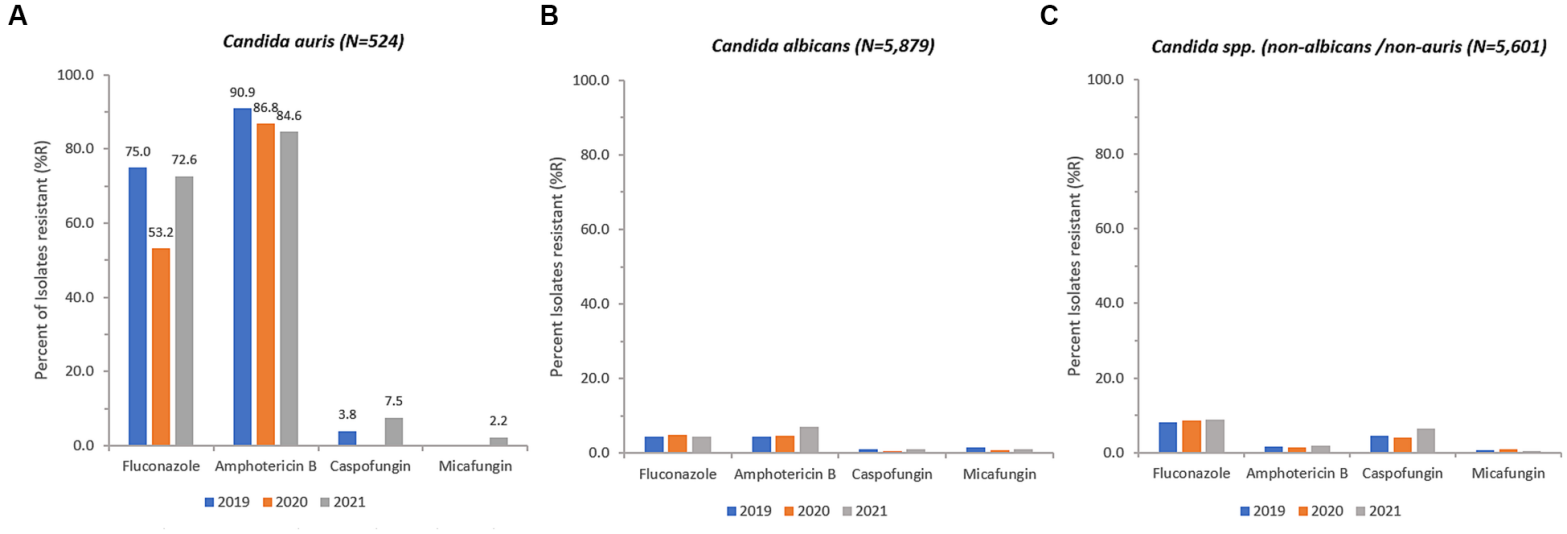
Annual trends for percentage of isolates resistant (% *R*) for *C. auris* **(A)**, *C. albicans* **(B)**, and *Candida* spp. (non-albicans/non-auris) **(C)**, 2018–2021.

Antifungal susceptibility testing data was available for 514 out of 809 (64.8%) non-duplicate *C. auris* isolates (fluconazole 480/809, 59.3%; amphotericin B 423/809, 52.3%; caspofungin 454/809, 56.1%; anidulafungin 11/809, 1.4%; micafungin 449/908, 55.5%). During the surveillance period *C. auris* resistance levels remained consistently high across all classes of antifungals used. *C. auris* in this population remains highly resistant to Azoles (fluconazole, 72.6% 2021) and rates have remained consistently high since 2019. Echinocandin resistance has now emerged and is increasing annually, from 3.8% (2019) to 7.5% (2021) for caspofungin, and from 0% (2019) to 2.2% (2021) for micafungin (Figure 8).

Resistance of *C. auris* to fluconazole was as high as 77.5% and 75.5% in isolates from skin and soft tissue, and respiratory tract, respectively, whereas fluconazole resistance was lower in isolates from urine (62.9%) and blood (64.2%). Resistance of *C. auris* to amphotericin B was highest in urine (87.2%), followed by respiratory tract isolates (85.1%), blood (84.8%), and skin and soft tissue (81.1%). Resistance of *C. auris* to caspofungin and micafungin ranged from 4.2% (blood) to 9.3% (urine), and 0% (blood) to 4.2% (urine), respectively.

Overall, 245 *C. auris* isolates out of 514 (47.67%) were MDR (≥ 2 antifungal classes resistant), including 20 isolates (3.89%) that were XDR (3 classes resistant, but one antifungal agent still susceptible), including 6 isolates (1.17%) that were PDR (resistant to all substances/all classes tested). The proportion of multidrug resistant *C. auris* isolates was 31.8% (14/44, 2019) and 28.4% (36/127, 2020), and increased in 2021 to 43.7% (150/343, 2021).

#### MIC distribution

**Figure 9:**
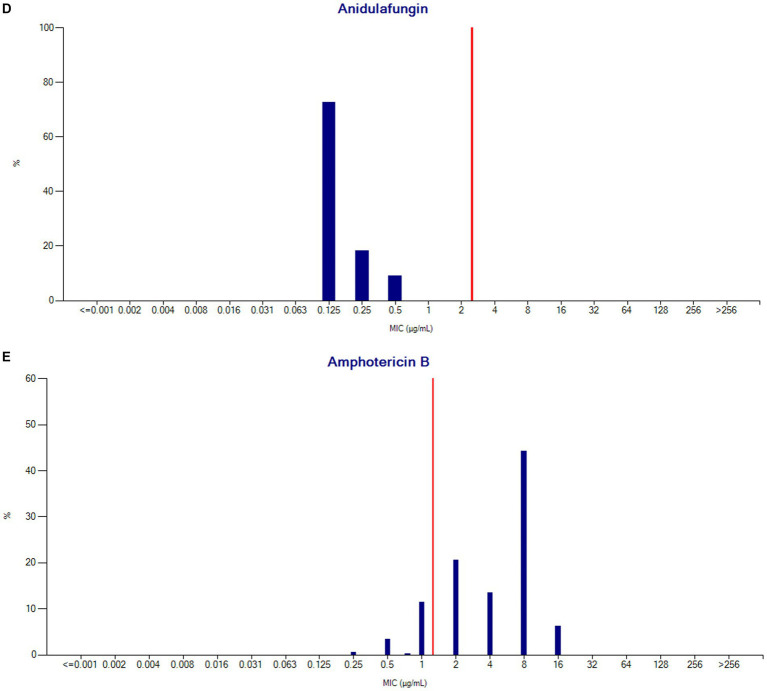
**(A)** Fluconazole MIC distribution (*n* = 480). **(B)** Caspofungin MIC distribution (*n* = 454). **(C)** Micafungin MIC distribution (*n* = 449). **(D)** Anidulafungin MIC distribution (*n* = 11). **(E)** Amphotericin B MIC distribution (*n* = 423).

MIC % RIS distributions were calculated for the collection of *C. auris* isolates based on the CDC tentative breakpoint recommendations and are presented below in Table 4 and Figures 9A–E.

**Table 4:** % RIS distribution for *Candida auris* isolates.

Antibiotic name	Breakpoints	Number	% *R*	% *I*	% *S*	% *R* 95% C.I.	% *S* 95% C.I.	MIC90
Fluconazole	*S* ≤ 16	480	67.92	0.00	32.08	63.5–72.0	28.0–36.5	256
	*R* ≥ 32							
Caspofungin	*S* ≤ 1	454	5.29	0.00	94.71	3.5–7.9	92.1–96.5	0.5
	*R* ≥ 2							
Micafungin	*S* ≤ 2	449	1.56	0.00	98.44	0.7–3.3	96.7–99.3	0.25
	*R* ≥ 4							
Anidulafungin	*S* ≤ 2	11	0.00	0.00	100.00	0.0–32.1	67.9–100	0.25
	*R* ≥ 4							
Amphotericin B	*S* ≤ 1	423	85.34	0.24	14.42	81.5–88.5	11.3–18.2	8
	*R* ≥ 2							

## Discussion

The growth in hospital sites reporting *Candida auris*, from only 2 centers in the first year to more than 34 sites towards the end of the study period, representing all 7 Emirates demonstrates considerable concern about *C. auris*. There is increased alertness across the country of the importance of antimicrobial resistance surveillance and mitigation.

The first cases of *C. auris* in UAE were detected in 2018. Since then, we have seen an alarming increase of *C. auris* isolations to *n* = 641 in 2021, especially in Abu Dhabi and Dubai. This increase is consistent with global reports of rising *C. auris* burden (56, 57). The COVID 19 pandemic does not seem to have impacted the dissemination of *C. auris*, and may have exacerbated it (58, 59). Nearly 50% of the patients were in intensive care and length of stay for these patients was extended by 19.5 days compared with patients infected with other *Candida* spp. Crude mortality at 27.5% (blood culture isolates: 36.1%) was similar to that for other *Candida* spp. and lower than seen in other countries (45% for blood culture isolates) (21).

*C. auris* is usually resistant to fluconazole and often to other antifungal medications (azoles, polyenes, and echinocandins). Multidrug-resistant and even pandrug-resistant *C. auris* isolates have also been described, which limits us to fewer and fewer treatment options (60–62). In this study, resistance rates of *C. auris* were high (fluconazole, 72.6% 2021, amphotericin B, 84.6% 2021), with the emergence of caspofungin and micafungin resistance in 2021, which is of great concern.

*C. auris* breakpoints are currently tentative. EUCAST will soon publish epidemiological cut-offs based on a global collection of isolates from which they have removed multiple epidemic or outbreak strains to minimise bias. Testing for fluconazole susceptibility shows very variable MICs, partly because of up-regulation of efflux pumps. These testing limitations may drive EUCAST to simply recommend that fluconazole is not used for *C. auris* infections, as they currently do for *C. glabrata* infections. There is general agreement that the tentative CLSI (and CDC) breakpoint for fluconazole is too high, and our finding that 27.4% of isolates were apparently susceptible to fluconazole aligns with this concern. There are also concerns about the breakpoint cut-off for amphotericin B as it bisects the wild type distribution, leading to uncertainty for MICs immediately above or below the breakpoint; an issue also described with Sensititre YeastOne testing (63). Although amphotericin resistance is high in our study, this may be an overestimation of resistance related to the susceptibility methods currently used, as highlighted in other studies (64, 65). Although we have detailed the *C. auris* breakpoints for the UAE for the first time, it is likely that new data and breakpoints will emerge.

There are no official guidelines for the management of *C. auris* infection in terms of an optimal antifungal agent(s) with dosing and duration regimen since CLSI/EUCAST breakpoints for this pathogen are yet to be defined (10, 27, 66). Echinocandins remain the first line therapy for *C. auris* infection, however as demonstrated by our data, resistance to all three main classes of antifungal agents remains a rising problem. Patients should be monitored closely to detect therapeutic failure and/or the development of resistance during their therapy (66).

The increasing trend of *C. auris* detection is suggestive of continued *C. auris* circulation predominately in hospitals. Thus infection control measures are critical to prevent continued dissemination. Such infection control measures could include better adherence to hand hygiene, appropriate use of transmission-based precautions based on setting, cleaning and disinfecting the patient care environment and reusable equipment with recommended products, communication about patient’s *C. auris* status when patient is transferred, screening contacts of newly identified case patients to identify *C. auris* colonization, and laboratory surveillance of clinical specimens to detect additional cases (67). Newly described approaches include UV-C light inactivation of *C. auris*, re-formulation of chlorhexidine for superficial use and silver nanoparticles as examples (68–71).

In the MENA region, *C. auris* has been reported from only six countries. Since genomic studies are lacking in the UAE, it was not possible to ascertain their similarity with *C. auris* clades from other geographic areas. Additional extensive research is needed on *C. auris* in the UAE to provide insight into its genetic epidemiology. Moreover, risk factors and methods of transmission need to be exhaustively identified to guide measures for prevention and to control the spread of the pathogen.

In conclusion, the emergence of *C. auris* poses a global health threat primarily to hospitalized and critically ill patients and should be met with a call for urgent action given its resistant patterns to various classes of antifungals. Our analysis of the national *C. auris* AMR surveillance data provides insights into the evolving patterns of disease and antimicrobial resistance in the UAE. The findings highlight the need for a continued surveillance program, particularly genomic epidemiological surveillance, to guide the continued AMR monitoring and active intervention and control measures to address the growing threat of antibiotic resistance. Furthermore continued *C. auris* circulation in hospitals requires enhanced infection control measures to prevent continued dissemination.

## Data availability statement

The national AMR Surveillance database managed by the UAE Ministry of Health and Prevention (MOHAP) contains confidential health information, and as such can only be made available upon reasonable request from the UAE Ministry of Health and Prevention (https://mohap.gov.ae).

## Ethics statement

Ethical approval for this study was provided by the Ministry of Health and Prevention Research Ethics Committee (MOHAP/DXB-REC/J.J.J./No. 86/2023), Dubai Scientific Research Ethics Committee (DSREC-GL17-2023), and Abu Dhabi Health Research and Technology Ethics Committee (DOH/ZHCD/2023/1316).

## Author contributions

DE, JT, NA, GM, CM, AS and the UAE AMR Surveillance Consortium: conceptualization and data collection. AO, DE, PSN, and JT: formal analysis. AO, AS, JT, NA, AA, DD, FA, GM, CM, and DE: data interpretation and manuscript review and editing. DE and JT: manuscript preparation. All authors contributed to the article and approved the submitted version.

## Figures and Tables

**Table:** 

**Nr.**	**Name**	**Institution**
1	Abiola Senok	College of Medicine, Mohammed Bin Rashid University of Medicine and Health Sciences, Dubai
2	Adnan Alatoom	Sheikh Shakhbout Medical City (SSMC), Abu Dhabi
3	Agnes-Sonnevend-Pal	University of Pécs, Pécs, Hungary
4	Ahmed Abdulkareem Al Hammadi	Tawam Hospital, Al Ain
5	Ahmed Elhag Ahmed	UAE University, College of Medicine and Health Sciences, Al Ain
6	Ahmed F. Yousef	Department of Biology, Center for Membranes and Advanced Water Technology, Khalifa University, Abu Dhabi
7	Alaa MM Enshasy	Dubai Health Authority, Dubai
8	Amal Mubarak Madhi	Abu Dhabi Public Health Center, Abu Dhabi
9	Amna AlBlooshi	Purelab, Al Ain
10	Andreas Podbielski	University Hospital Rostock, Rostock, Germany
11	Anju Nabi	Dubai Academic Health Corporation (DAHC), Dubai
12	Anup Shashikant Poddar	Al Sharq Hospital, Fujairah
13	Arun Kumar Jha	Danat Al Emarat Hospital, Abu Dhabi
14	Ayesha Abdulla Al Marzooqi	Abu Dhabi Public Health Center, Abu Dhabi
15	Bashir Aden	Khalifa University, Abu Dhabi
16	Carole Ayoub Moubareck	College of Natural and Health Sciences, Zayed University, Dubai
17	Dean Everett	Department of Pathology and Infectious Diseases, College of Medicine, Khalifa University, Abu Dhabi
18	Deeba Jafri	Purelab, Sheikh Khalifa Medical City, Ajman
19	Duckjin Hong	Sheikh Khalifa Specialty Hospital (SKSH) RAK
20	Emmanuel Fru Nsutebu	Sheikh Shakhbout Medical City, Abu Dhabi
21	Farah Ibrahim Al-Marzooq	United Arab Emirates University, Al Ain
22	Fatima Al Dhaheri	United Arab Emirates University, Al Ain
23	Fouzia Jabeen	Purelab, Sheikh Khalifa Hospital, Abu Dhabi
24	Francis Amirtharaj Selvaraj	Sheikh Khalifa Medical City (SKMC), Abu Dhabi
25	Ghada Abdel Wahab	Abu Dhabi Agriculture and Food Safety Authority, Abu Dhabi
26	Ghalia Abdul Khader Khoder	University of Sharjah, Sharjah
27	Gitanjali Avishkar Patil	NMC Specialty Hospital, Abu Dhabi
28	Godfred A. Menezes	Department of Medical Microbiology and Immunology, RAK Medical and Health Sciences University, Ras Al Khaimah
29	Hadayatullah Ghulam Muhammad	Emirates International Hospital, Al Ain
30	Hafiz Ahmad	RAK Hospital, Ras Al Khaimah
31	Hala Ahmed Fouad Ismail	PureLab, Al Qassimi Hospital, Sharjah
32	Hazim Khalifa	Department of Veterinary Medicine, UAE University, Al Ain
33	Husein Alzabi	Sheikh Khalifa General Hospital, Um al Quwain
34	Ibrahim Alsayed Mustafa Alhashami	Purelab, Al Qassimi Hospital, Sharjah
35	Imene Lazreg	University of Sharjah, Sharjah
36	Irfaan Akthar	Mediclinic City Hospital, Dubai
37	Jens Thomsen	Abu Dhabi Public Health Center, Abu Dhabi
38	John Stelling	WHONET, Boston, USA
39	Kaltham Ali Kayaf	Ministry of Climate Change & Environment (MOCCAE), Dubai
40	Kavita Diddi	Prime Hospital, Dubai
41	Krishnaprasad Ramabhadran	Burjeel Hospital, Abu Dhabi
42	Laila Al Dabal	Dubai Academic Health Corporation (DAHC, Dubai)
43	Laura Thomsen	University of Freiburg, Germany
44	Leili Chamani-Tabriz	Clemenceau Medical Center, Dubai
45	Madikay Senghore	Khalifa University, Abu Dhabi
46	Manal Abdel Fattah Ahmed	PureLab, Ras Al Khaimah
47	Maya Habous	Rashid Hospital, Dubai Academic Health Corporation, Dubai
48	Moeena Zain	American Hospital Dubai
49	Mohamud M. Sheek-Hussein	United Arab Emirates University, Al Ain
50	Monika Maheshwari	Al Zahra Hospital, Dubai
51	Monika Maheshwari	Medeor 24x7 Hospital, Dubai
52	Mubarak Saif Alfaresi	Zayed Military Hospital, Abu Dhabi
53	Mushtaq Khan	United Arab Emirates University, Al Ain
54	Najiba Abdulrazzaq	Al Kuwait Hospital, Emirates Health Services Establishment, Dubai
55	Nehad Nabeel Al Shirawi	Al Fujairah Hospital
56	Nesrin Helmy	Mediclinic Al Noor Hospital - Khalifa Street, Abu Dhabi
57	Pamela Fares Mrad	Abu Dhabi Public Health Center (ADPHC), Abu Dhabi
58	Pascal Frey	Berne University Hospital, Berne, Switzerland
59	Peter Nyasulu	Department of Global Health, Faculty of Medicine and Health Sciences, Stellenbosch University, South Africa
60	Prashant Nasa	NMC Specialty Hospital Al Nahda, Dubai
61	Rajeshwari T. A. Patil	Burjeel Medical City, Abu Dhabi
62	Rania El Lababidi	Dept. of Pharmacy Services, Cleveland Clinic Abu Dhabi
63	Ratna A. Kurahatti	NMC Royal Hospital Khalifa City A, Abu Dhabi
64	Riyaz Amirali Husain	Dubai Hospital, Dubai Academic Health Corporation, Dubai
65	Robert Lodu Serafino Wani Swaka	Sheikh Shakhbout Medical City, Abu Dhabi
66	Saeed Hussein	Erada Center for Treatment and Rehabilitation, Dubai
67	Sameh Soliman	University of Sharjah, Sharjah
68	Savitha Mudalagiriyappa	University Hospital Sharjah, Sharjah
69	Seema Oommen	Burjeel Medical City, Abu Dhabi
70	Shaikha Ghannam Alkaabi	Abu Dhabi Public Health Center, Abu Dhabi
71	Simantini Jog	Fakeeh University Hospital, Dubai
72	Simantini Jog	King’s College Hospital London Dubai Hills, Dubai
73	Siobhan O‘Sullivan	Khalifa University, Abu Dhabi
74	Somansu Basu	NMC Specialty Hospital, Al Ain
75	Stefan Weber	Purelab, Abu Dhabi
76	Sura Khamees Majeed	Al Gharbia Hospitals - Madinat Zayed Hospital
77	Syed Irfan Hussein Rizvi	Mediclinic City Hospital, Dubai
78	Tibor Pal	University of Pécs, Pécs, Hungary
79	Timothy Anthony Collyns	Tawam Hospital, Al Ain
80	Yassir Mohammed Eltahir Ali	Animal Wealth Sector, Abu Dhabi Agriculture and Food Safety Authority, Abu Dhabi
81	Yousuf Mustafa Naqvi	Department of Health Abu Dhabi (DoH), Abu Dhabi
82	Zahir Osman Babiker	Sheikh Shakhbout Medical City (SSMC), Abu Dhabi
83	Zulfa Omar Al Deesi	Latifa Maternity & Pediatric Hospital, Dubai

## Footnotes

^1^ https://www.whonet.org
