# Supplementary figures and images for: Emergence of highly resistant Candida auris in the United Arab Emirates: a retrospective analysis of evolving national trends
Source: Front Public Health. 2024 Jan 12;11:1244358. doi: 10.3389/fpubh.2023.1244358 (PMC10826512; doi:10.3389/fpubh.2023.1244358)

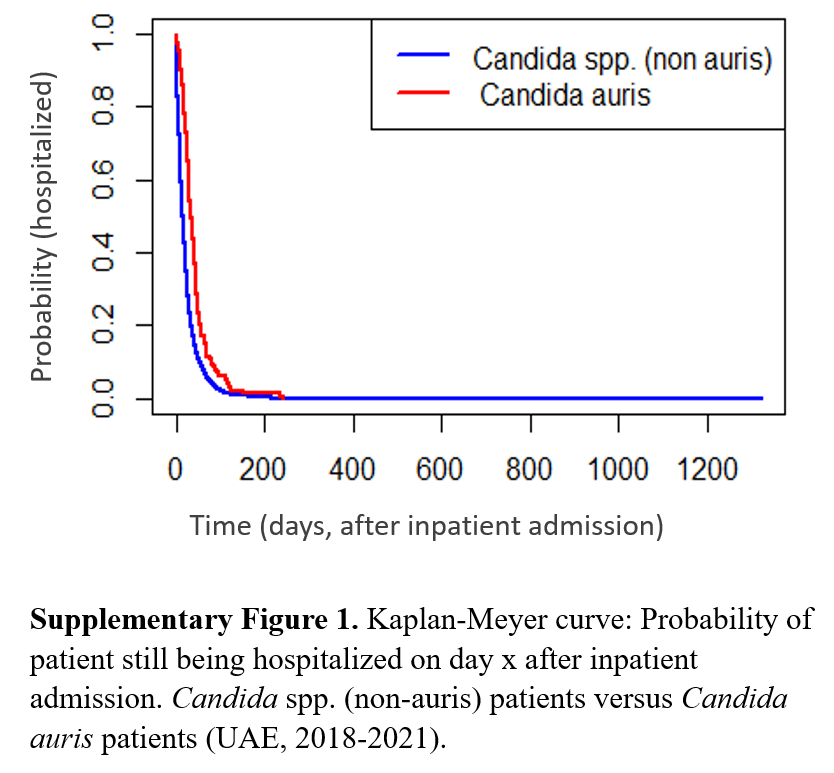

Supplement: Supplementary file 1 [file Image_1.tif]
